# Expression and possible functions of a horizontally transferred glycosyl hydrolase gene, *GH6-1*, in *Ciona* embryogenesis

**DOI:** 10.1186/s13227-023-00215-x

**Published:** 2023-07-11

**Authors:** Kun-Lung Li, Keisuke Nakashima, Kanako Hisata, Noriyuki Satoh

**Affiliations:** 1 Marine Genomics Unit, Okinawa Institute of Science and Technology Graduate University, Onna, Okinawa 904-0495 Japan; 2 Present Address: Institute of Cellular and Organismic Biology, Academia Sinica, Taipei City, 115 Taiwan

**Keywords:** Tunicates, Horizontally transferred genes, Glycosyl hydrolase family-6 *GH6* gene, Expression and function in *Ciona* embryogenesis

## Abstract

**Background:**

The Tunicata or Urochordata is the only animal group with the ability to synthesize cellulose directly and cellulose is a component of the tunic that covers the entire tunicate body. The genome of *Ciona intestinalis* type A contains a cellulose synthase gene, *CesA,* that it acquired via an ancient, horizontal gene transfer. *CesA* is expressed in embryonic epidermal cells and functions in cellulose production. *Ciona* CesA is composed of both a glycosyltransferase domain, GT2, and a glycosyl hydrolase domain, GH6, which shows a mutation at a key position and seems functionless. Interestingly, the *Ciona* genome contains a glycosyl hydrolase gene, *GH6-1,* in which the GH6 domain seems intact. This suggests expression and possible functions of *GH6-1* during *Ciona* embryogenesis. Is *GH6-1* expressed during embryogenesis? If so, in what tissues is the gene expressed? Does *GH6-1* serve a function? If so, what is it? Answers to these questions may advance our understanding of evolution of this unique animal group*.*

**Results:**

Quantitative reverse transcription PCR and in situ hybridization revealed that *GH6-1* is expressed in epidermis of tailbud embryos and in early swimming larvae, a pattern similar to that of *CesA.* Expression is downregulated at later stages and becomes undetectable in metamorphosed juveniles. The *GH6-1* expression level is higher in the anterior-trunk region and caudal-tip regions of late embryos. Single-cell RNA sequencing analysis of the late tailbud stage showed that cells of three clusters with epidermal identity express *GH6-1*, and that some of them co-express *CesA*. TALEN-mediated genome editing was used to generate *GH6-1* knockout *Ciona* larvae. Around half of TALEN-electroporated larvae showed abnormal development of adhesive papillae and altered distribution of surface cellulose. In addition, three-fourths of TALEN-electroporated animals failed to complete larval metamorphosis.

**Conclusions:**

This study showed that tunicate *GH6-1*, a gene that originated by horizontal gene transfer of a prokaryote gene, is recruited into the ascidian genome, and that it is expressed and functions in epidermal cells of ascidian embryos. Although further research is required, this observation demonstrates that both *CesA* and *GH6-1* are involved in tunicate cellulose metabolism, impacting tunicate morphology and ecology.

**Supplementary Information:**

The online version contains supplementary material available at 10.1186/s13227-023-00215-x.

## Background

The Tunicata or Urochordata is one of three chordate taxa, together with the Cephalochordata and Vertebrata. Although the Tunicata and Vertebrata have a sister relationship [1–3], tunicates are unique among animal groups since they are enclosed by a cellulose-containing tunic and they are able to synthesize cellulose by themselves [4, 5]. The ability to utilize cellulose to form the tunic has strongly influenced their lifestyle, morphology, physiology, and ecology [5–8], leading to a peculiar animal lineage or phylum [9].

Tunicate cellulose synthesis relies on a cellulose synthase gene, *CesA* [10–14]. Molecular phylogeny shows that tunicate *CesA* was likely derived by horizontal transfer of a prokaryote *CesA* into the genome of tunicate ancestor*,* although the bacterial source has not been identified yet [4, 10, 12, 15, 16]. Intriguingly, tunicate CesA is composed of not only a glycosyl transferase (GT2) domain (Pfam: PF13641) at the N-terminus of the protein, but also a glycosyl hydrolase (GH6) domain at the C-terminus (Pfam: PF01341) [10, 12]. The biological importance and enzymatic activity of the GH6 domain are not yet known. In addition, the conserved ‘signature 2’ of GH6 (PROSITE PS00656) in tunicate CesAs lacks an important aspartic acid, which corresponds to the catalytic center of well-studied fungal GH6 cellulase [10, 12, 17]. Therefore, this GH6 domain is likely functionless [16].

Expression and function of *CesA* during *Ciona* embryogenesis have been well studied [12, 14, 18]. This gene is expressed in differentiating epidermal cells of tailbud embryos and hatched swimming larvae [12, 14]. A transposon-mediated mutation of *Ciona*, named *swimming juvenile* (*sj*), demonstrated that loss of *CesA* function results in failure of cellulose synthesis in embryonic epidermal cells, and also affects metamorphosis of swimming larvae to sessile juveniles [14]. A transcription factor gene, *Tfap2* (*AP2*), is expressed in embryonic epidermis [18, 19]. The 5′ regulatory region of *Ci-CesA* contains an AP2 binding site (GCCTGCGGGC) between bases − 431 and − 450 and deletion of this site results in loss of *Ci-CesA* expression in embryonic epidermis [18]. This raises the possibility that the bacterial *CesA* gene was recruited into an ancient gene regulatory network (GRN) of the tunicate ancestral genome, which permitted expression in larval epidermis.

Animals from many phyla have a glycosyl hydrolase gene of the GH9 family [20–22], that functions in digestion of cellulose consumed as food. However, no known animals or plants have a gene from the GH6 family. Our previous study discovered a gene that encodes another conserved GH6 domain in the genome of *Ciona intestinalis* and several other tunicates [4, 16]. The gene was named *GH6-1* to distinguish this domain from the GH6 domain present in CesA [16]. In contrast to the GH6 of CesA, many tunicate GH6-1 proteins retain the aspartic acid in the active site, suggesting retained function [16]. Molecular phylogeny shows that *GH6-1* originated from horizontal gene transfer, very likely of bacterial origin [16]. Therefore, we sought to determine whether *GH6-1* is expressed in ascidian embryos, and if so, where. What function(s) does *GH6-1* exhibit? In *Ciona intestinalis* type A, *CesA* is located on the long arm of chromosome 7 (the Ghost Database: KY21.Chr7.453 or KY.Chr7.448), whereas *GH6-1* is located on the short arm of chromosome 3 (the Ghost Database: KY21.Chr3.438 or KY.Chr3.452) [23–25]. Is there any relationship in expression and function of these two horizontally transferred genes? This study addressed these questions.

## Results

### Expression of *GH6-1 *and *CesA *during early *Ciona* development

**Fig. 1:**
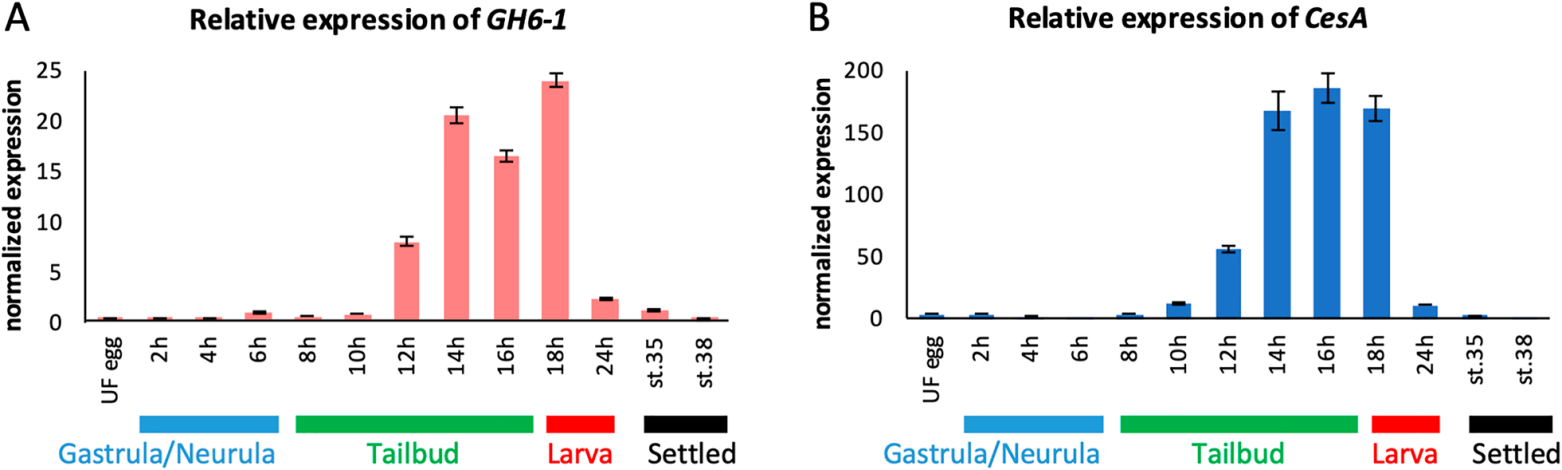
Quantitative changes in expression levels of *GH6-1* (left) and *CesA* (right) during early development of *Ciona intestinalis* type A. The X-axis shows developmental time in hours and stages. The Y-axis shows gene expression levels. The expression level of each gene was first normalized to a ubiquitously expressed *GAPDH* (glyceraldegyde-3-phosphate dehydrogenase) and then expression at 6 h post fertilization (hpf) was set as 1 for normalization. Error bars indicate the standard error of three experimental replicates. Developmental staging follows the TUNICANATO website [44, 57]

Temporal and spatial expression of *GH6-1* and *CesA* in *Ciona* embryos, larvae, and newly metamorphosed juveniles were examined by reverse transcription quantitative polymerase chain reaction (RT-qPCR) and whole-mount in situ hybridization (Fig. 1). Expression of *GH6-1* was undetectable or nearly so until early tailbud stage (10 h post fertilization (hpf) at 18 °C) (Fig. 1A). Expression became evident at mid tailbud stage (12 hpf) and increased until late tailbud stages (14 and 16 hpf). Hatched larvae (18 hpf) showed the highest level of *GH6-1* expression, but it was then downregulated and became undetectable in late larvae (24 hpf) and metamorphosed/settled juveniles (stages 35 and 38).

The temporal expression profile of *CesA* was also determined for comparison with that of *GH6-1* (Fig. 1B). In accordance with results of a previous study [12], expression of *CesA* was detected in tailbud embryos and larvae (12–18 hpf). Its expression level began to rise at the mid tailbud stage and reached its zenith at late tailbud stage (16 hpf). Newly hatched larvae (18 hpf) also showed high *CesA* expression, but it was greatly reduced in 24-hpf larvae and settled juveniles (stages 35 and 38). These results document similar temporal expression profiles of *GH6-1* and *CesA* during *Ciona* embryogenesis.

**Fig. 2:**
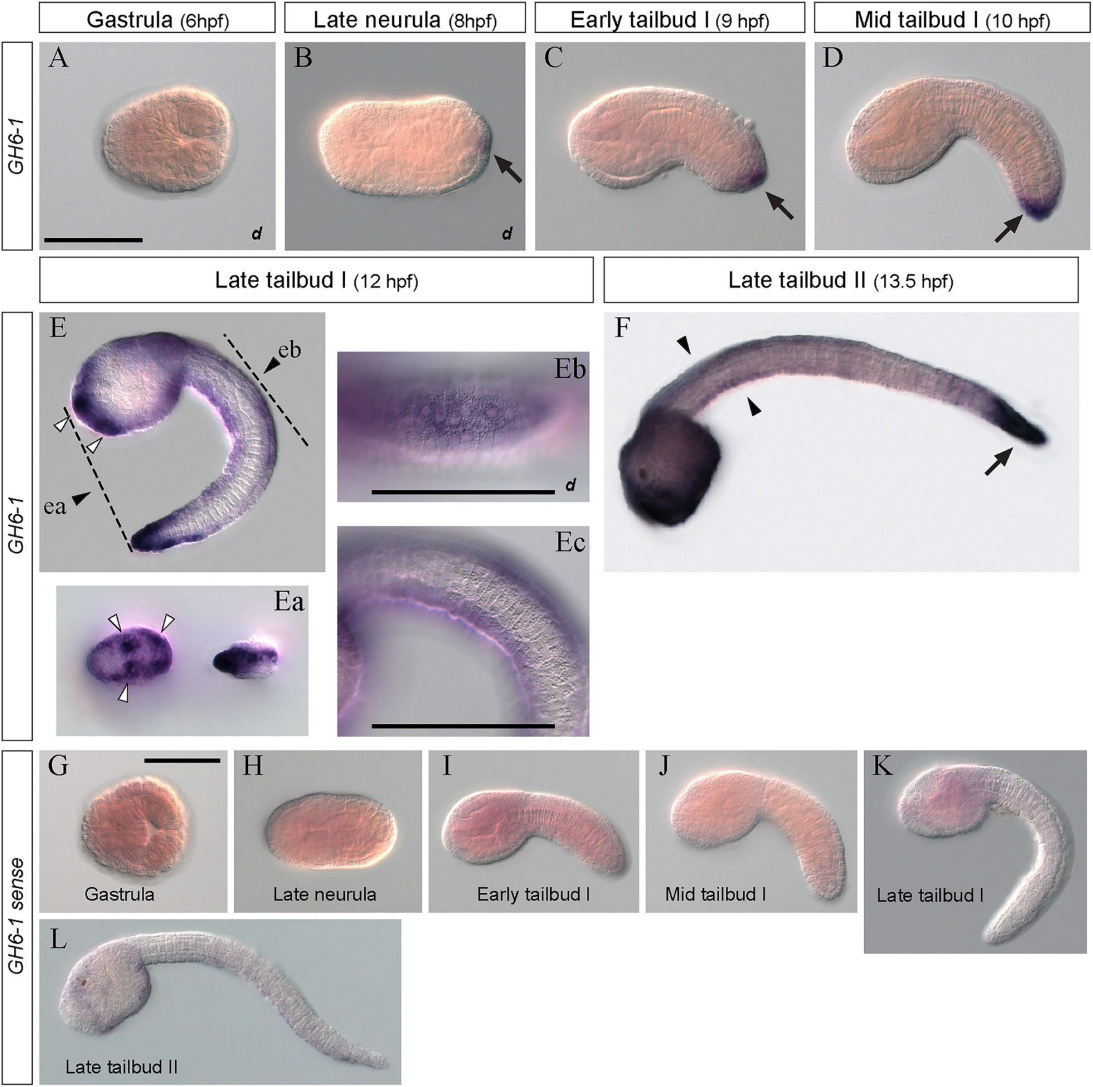
In situ hybridization of spatial expression of *GH6-1* in *Ciona* embryos*.* **A** The hybridization signal was not observed in gastrula stage (6 hpf). **B** The *GH6-1* signal in epidermal cells is first observed at the tail-tip region of late neurulae (arrow; 8 hpf) followed by **C** early (arrow; 9 hpf) and **D** mid tailbud stages (arrow; 10 hpf). **E**,** F** Expanded signals of *GH6-1* are evident in epidermal cells at (**E**) late tailbud I stage (12 hpf) and (**F**) late tailbud II stage (13.5 hpf). Panels **Ea**, **Eb**, and **Ec** show enlargement of the area shown in **E**. **Ea** The specimen viewed from anterior (left) and posterior side (right). White arrowheads show regions corresponding to adhesive papilla primordia; **Eb** viewed from the dorsal side, and **Ec** from the side. **F** Late tailbud stage (13.5 hpf). Arrows show a strong signal at tail-tip epidermis, while arrowheads show moderate signal along tail midline epidermis. **G**–**L** Control embryos treated with sense riboprobe show undetectable level of signal. **G** Gastrula; **H** late neurula; **I** early-to-mid tailbud; **J** mid tailbud; **K** late tailbud I; **L** late tailbud II. **A**–**E**, **Ea**, and **F** are at the same magnification as panel **A**, in which a scale bar represents 100 μm. Scale bars in **Eb** and **Ec** represent 100 μm. **G** Shows a scale bar of 100 μm; **H**–**L** Are at the same magnification as **G**. The *d* footnote in **A**, **B**, and **Eb** denotes a dorsal view

Spatial expression of *GH6-1* in *Ciona* embryos was examined by whole-mount in situ hybridization (Fig. 2). The hybridization signal of *GH6-1* mRNA was not detected in gastrulae (Fig. 2A, 6 hpf). It first appeared in the epidermis of future tail tip of late neurulae (Fig. 2B, 8 hpf). The tail tip signal persisted in early (Fig. 2C, 9 hpf) and mid tailbud stages (Fig. 2D, 10 hpf). Later, at late tailbud stage I, dorsal and ventral midline epidermis of the tail and many trunk epidermal cells also showed signals (Fig. 2Ea–c, 12 hpf). In particular, three regions of anterior trunk showed stronger signals (open arrows in Fig. 2E, Ea). These regions likely correspond to adhesive papilla primordia (Fig. 2Ea). At late tailbud stage II (13.5 hpf), a strong signal was observed in epidermis of the trunk and tail tip (arrow, Fig. 2F) while signal in the middle portion of the tail was not so strong (arrowheads, Fig. 2F). Control embryos treated with a sense riboprobe showed no signals during the stages examined (Fig. 2G–L).

**Fig. 3:**
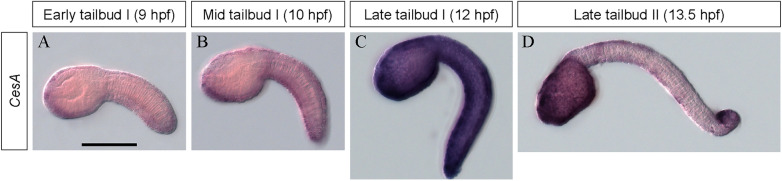
In situ hybridization of the *CesA* gene in *Ciona* embryos. Signals for *Ciona CesA* expression become detectable at **A** early tailbud (9 hpf), **B** mid tailbud (10 hpf), **C** late tailbud I (12 hpf) and **D** late tailbud II (13.5 hpf). Signals appear in epidermis more ubiquitously than expression of *GH6-1*. The scale bar in panel A represents 100 μm and applies to all panels

Spatial expression of *CesA*, another horizontally transferred gene, was also examined and compared to the *GH6-1* expression profile (Fig. 3). Hybridization signals of *CesA* mRNA appeared at early and mid-tailbud stages (Fig. 3, 9 and 10 hpf). Strong signals appeared in all epidermis at late tailbud I stage (Fig. 3, 12 hpf). At late tailbud stage II, the signal became weak throughout the entire tail region (Fig. 3, 13.5 hpf). Therefore, *CesA* and *GH6-1* showed a similar spatial expression profile. A difference occurred at late tailbud stages. *CesA* signals were broader, whereas *GH6-1* signals were strong in the most anterior and caudal regions of embryos.

### Expression of* GH6-1* and *CesA* in embryonic epidermis at the single-cell level

**Fig. 4:**
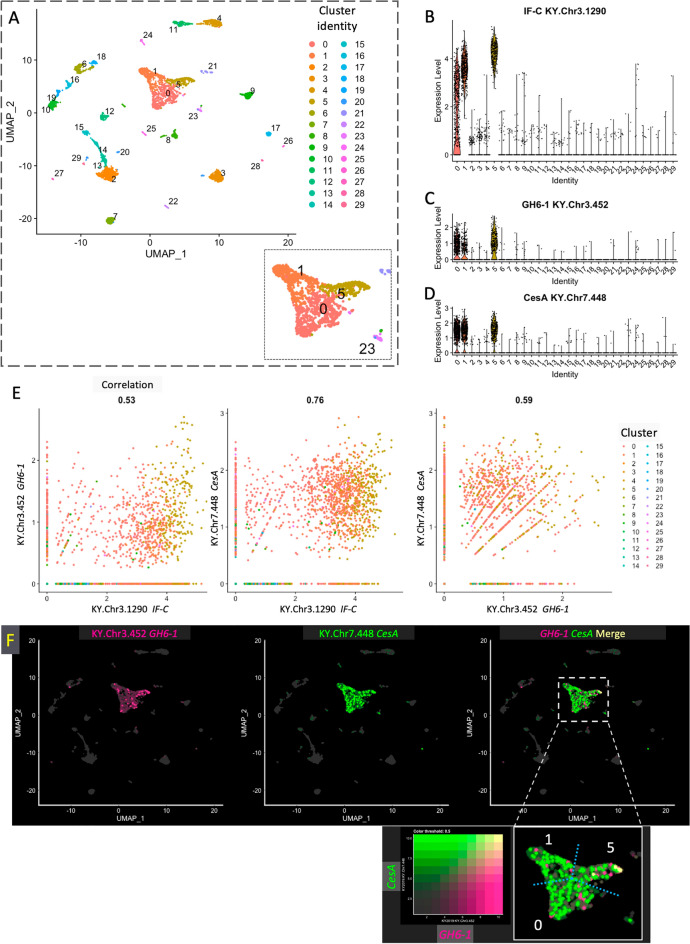
Single-cell transcriptome analysis showed that *Ciona GH6-1* and *CesA* expression correspond to epidermal cell identity. **A** A dimension-reduction plot shows a representation of late tailbud stage I embryonic cells, separated into 30 clusters (numbered 0 to 29). Dimensions were reduced by the uniform manifold approximation and projection (UMAP) technique in the Seurat package. Each dot represents the transcriptome of a single cell. Cells in the same cluster have similar gene expression profiles. Numbers and color labels denote cluster identities. **B**–**D** Violin plots showing expression levels of three genes. X-axis: cell cluster identifier. Y-axis: normalized expression level of each gene. **B** The *IF-C* gene (KY.Chr3.1290) was selected as an epidermal marker of cell identity of clusters. It was highly expressed in cells in clusters No. 1 and 5 and to a lesser extent in cluster 0. **C**, **D** *GH6-1*-expressing cells and *CesA*-expressing cells were mostly identified in clusters 0, 1, and 5. **E** Scatterplots showing relationships of normalized expression of *IF-C*, *GH6-1*, and *CesA*. Each dot represents the transcriptome of a cell and cells are colored by cluster identity. The X- and Y-axes represent normalized expression levels. Pearson correlation between the two features is displayed above each plot. **F** UMAP-dimension-reduction plots showing normalized expression levels of *GH6-1* and *CesA* genes. Although expression of these two genes appeared mostly in the same cell clusters 0, 1, and 5, only a few cells show high expression of both genes (yellow dots). The enlarged, dashed rectangular area is shown as an insert at the bottom-right. Note that KY gene models (2019 version, KY.ChrX.yyyy) were used in the original analysis and the corresponding KY21 gene models (the latest version) are described in the main text

As described above, *GH6-1* is expressed in epidermal cells of late tailbud embryos, as in the case of *CesA*. However, this does not necessarily mean that all individual cells express both genes coincidentally. To examine this question, we took advantage of single-cell RNA sequencing (scRNA-seq) data of *Ciona* late tailbud stage I embryos provided by [26]. Based on the criterion that cells of a given cluster share similar gene expression patterns, we categorized constituent cells of late tailbud embryos into 30 clusters, numbered from 0 to 29 (Fig. 4A). To identify the epidermal-cell cluster, an intermediate filament protein gene, *IF-C* (KY21.Chr3.1271/KY.Chr3.1290 from the Ghost database; see also LOC100175966 on the NCBI Gene database) [26, 27], was selected as a marker of epidermal cells. The *IF-C* was highly expressed in cells of clusters 0, 1, and 5, suggesting that these clusters possess properties of epidermis (Fig. 4B). Close localization of the three clusters in a UMAP plot supports the similarity of the three clusters (Fig. 4A, insertion). *GH6-1* was expressed in cells of clusters 0, 1 and 5 (Fig. 4C). Similarly, *CesA* was expressed in cells of the three clusters (Fig. 4D). The result coincides with that obtained by in situ hybridization analysis.

Correlation of normalized expression levels of *CesA* and *GH6-1* were examined with *IF-C* by cell plots (Fig. 4E). Correlation coefficients between *GH6-1* and *IF-C* (Fig. 4E, left)*,* between *CesA* and *IF-C* (Fig. 4E, middle)*,* and between *CesA* and *GH6-1* (Fig. 4E, right) were 0.53, 0.76, and 0.59, respectively. This indicates that expression of *CesA* and *IF-C* was more highly correlated, than those of *GH6-1* and *IF-C* and of *CesA* and *GH6-1,* which were similar.

Furthermore, *GH6-1* and *CesA* expression levels were plotted on dimension-reduction plots (Fig. 4F). Plots of cells expressing *GH6-1* (Fig. 4F, left) and *CesA* (Fig. 4F, middle) showed that the number of constituent cells with *CesA* expression was larger than the number expressing *GH6-1.* In addition, cells with *CesA* expression were distributed more broadly than those with *GH6-1.* This may be correlated with results mentioned above. That is, expression co-efficiency was higher between *CesA* and *IF-C* than between *GH6-1* and *IF-C.* Double blotting of *GH6-1-*expressing cells with *CesA-*expressing cells revealed that a few cells showed high expression of both genes simultaneously (Fig. 4F, right; bottom-right inset). Such cells were seen in cluster 5. On the other hand, many cells expressed only *CesA* (Fig. 4F, right). This result indicates that there are some epidermal cells in *Ciona* embryos that express *GH6-1* and *CesA* coincidently.

### Possible functions of *Ciona GH6-1*

To examine possible functions of *GH6-1* in *Ciona* embryos and larvae, we used TALEN-mediated genome editing to knockout *GH6-1* of *C. intestinalis* type A. Customized TALEN-expression plasmids were introduced into fertilized *Ciona* eggs by electroporation. Nuclease activity of TALEN (*Fok*I part) depends upon heterodimerization of both nuclease subunits [28, 29]. When we introduced single-sided-TALEN plasmids (which alone cannot produce functional nuclease dimers) into specimens, there was no change of genomic sequence around the designed target (see Methods, no change in sequences in any of the 8 amplicons). In addition, in a pioneer experiment, genome-editing efficiency of two TALEN pairs was evaluated by sequencing the corresponding genomic DNA amplicons (see Methods). TALEN pair No.1 had an editing efficiency of 57% (8 of 14 amplicons showed the edited sequence) and TALEN pair No.2 had an efficiency of 63% (10 of 16 amplicons were edited). Therefore, TALEN pair No.2 was selected for the rest of the study. Binding sites of this pair were nucleotides 429–478 of the assumed coding sequence of *GH6-1*, and cut sites were around coding nucleotide 453. We observed a higher proportion of phenotypically affected individuals (described below).

Beyond introducing paired TALEN plasmids, three control groups were created. The first group comprised dechorionated eggs that were passed through mannitol solution washes (see Methods section) without electroporation. The second group comprised eggs that received an mVenus plasmid containing the promotor of *Ciona* forkhead gene, *Ci-fkh,* and monomeric Venus fluorescent protein gene. The third group comprised eggs that received a TALEN plasmid that encodes a single TALEN subunit that was not expected to form functional nuclease dimers.

**Fig. 5:**
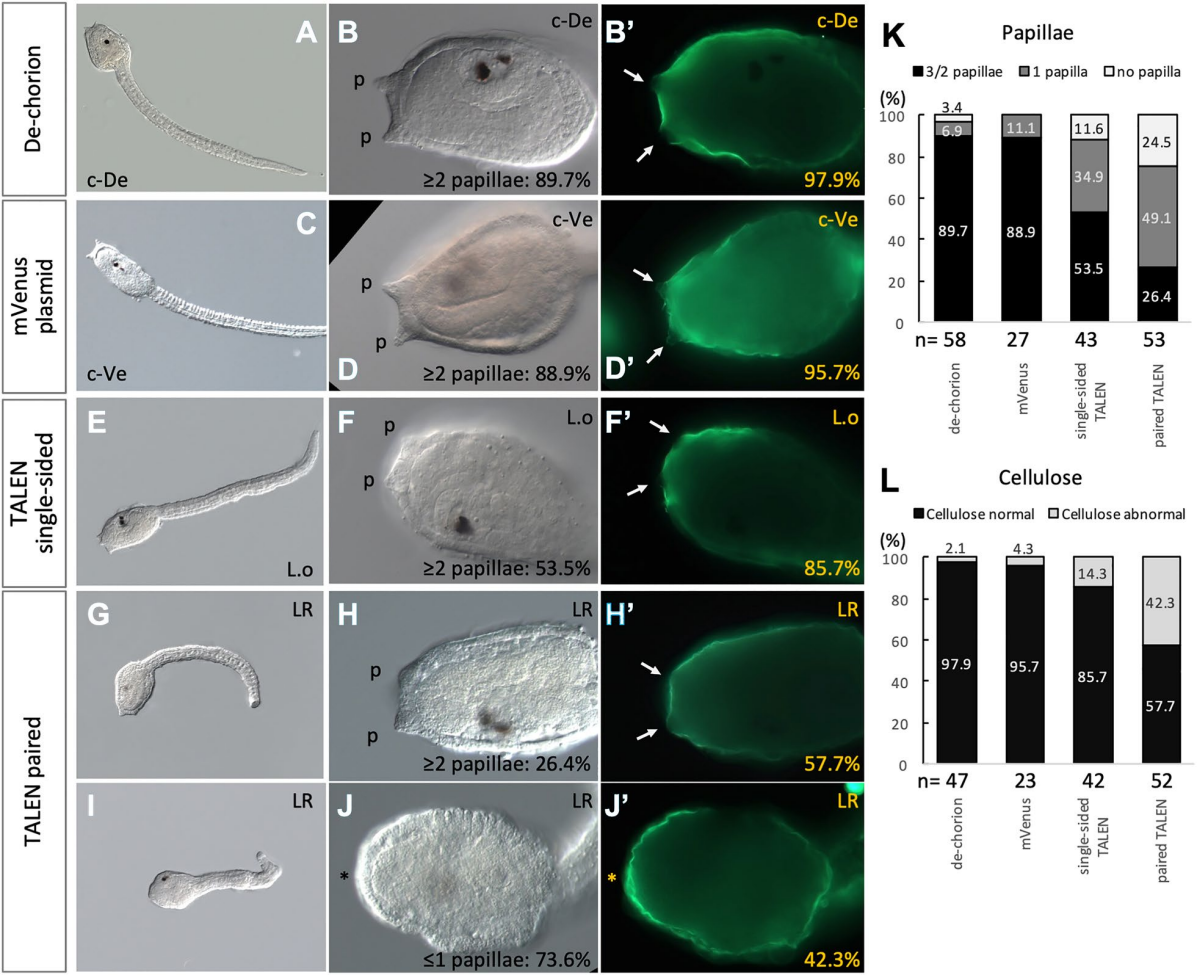
*GH6-1* knockout by TALEN-mediated genome editing affected *Ciona* larval development. **A**, **B** Control larvae developed from dechorionated eggs. **C**, **D** Control larvae developed from eggs electroporated with mVenus plasmids. **E**, **F** Control larvae developed from eggs electroporated with single-sided TALEN. **G**–**J** Experimental larvae developed from eggs electroporated with paired TALEN plasmids. While most control larvae showed adhesive papillae (p in **B**–**H**) and regionally reduced cellulose (arrows in **B**′–**F**′), many larvae of *GH6-1* TALEN knockout failed to form adhesive papillae (asterisk in **J**) and show a strong cellulose signal all over the anterior epidermis (**J**′). **K**, **L** Percentages of larvae of each phenotype. **K** Larvae were grouped as: 3 or 2 papillae, 1 papilla, no papilla. **L** Cellulose normal: surface cellulose was found at the larval tunic outside the epidermis, while cellulose signal was reduced around papillae in the anterior trunk. Cellulose abnormal: surface cellulose exists, but there is no local reduction of cellulose signal strength around papillae. **A**–**J** Are DIC images; **B**′, **D**′, **F**′, **H**′ and **J**′ are green fluorescence channels showing cellulose after CBM-GFP staining. c-De, control-dechorionated group. c-Ve, control-mVenus group. L.o, Only the left TALEN plasmid was introduced during electroporation. LR: both of the left and right TALEN plasmids were introduced during electroporation. p: adhesive papillae. Asterisk sign (*): no papilla formation

When manipulated, embryos developed to the hatching larva stage (17.5–18 hpf at 18 °C). These control groups showed normal larval morphology, having three protruding adhesive papillae at the anterior end (Fig. 5A, B for dechorionation; Fig. 5C, D for mVenus plasmid electroporation; and Fig. 5E, F for single-sided TALEN electroporation). In contrast, TALEN-electroporated larvae showed several types of abnormality. We first observed abnormal development of papillae (Fig. 5I, J). Many of these larvae either had only one papilla (49.1%) or did not develop papillae at all (24.5%) (Fig. 5K). On the other hand, in the single-sided TALEN group (Fig. 5E, F), development of papillae was also moderately affected: 46.5% of the larvae showed only one or no papillae (Fig. 5K), and some papillae were shorter than usual. As already mentioned, the single-sided TALEN group showed no genomic sequence change around the *GH6-1* gene. It is possible that excessive exotic protein products driven by a strong promoter alone caused cellular stress or that binding of inactive TALEN proteins to genomic DNA affected normal gene expression, although further investigation would be required to clarify abnormal development in the single-sided-TALEN animals.

We observed altered cellulose distribution in TALEN-electroporated larvae. As described above, *GH6-1* is expressed in epidermal cells of *Ciona* embryos and larvae, where *CesA* is expressed as well, and scRNA-seq analyses showed that some cells expressed both genes coincidently. Since we questioned whether tunicate GH6-1 protein might participate in cellulose metabolism, we examined the existence and abundance of surface cellulose of the larval tunic, which normally is formed shortly before *Ciona* larvae hatch. Larvae were stained with Green Fluorescent Protein-linked carbohydrate binding modules (GFP-CBM) (Fig. 5B′–J′). In controls (Fig. 5B′ for dechorionation; Fig. 5D′ for mVenus plasmid electroporation; Fig. 5F′ for single-sided TALEN electroporation), larvae showed a cellulose signal on the larval tunic external to the epidermis. Notably, compared to other parts of trunk epidermis, a weaker cellulose signal was observed in regions surrounding papillae in epidermis of the anterior trunk (Fig. 5B′, D′, F′). All paired TALEN-electroporated larvae also showed cellulose on the larval tunic (Fig. 5H′, J′). Although about half of TALEN-electroporated larvae (57.7%) also showed a normal pattern of locally reduced cellulose at the papilla region (Fig. 5H′), epidermis of the most anterior part in nearly half of TALEN-electroporated larvae (42.3%) did not show any area with a reduced level of cellulose (Fig. 5J′). In other words, the anterior epidermis of these affected larvae showed a relatively stronger cellulose signal than the corresponding part of control larvae. A smaller portion (14.3%) of single-sided TALEN group larvae also showed the loss of local cellulose reduction. On the other hand, after dechorionation, embryos and larvae only produced a thin layer of larval tunic, which covered both the trunk and the tail. Although *GH6-1* is expressed along midlines of embryonic tails (Fig. 2), we could not find a clear difference or alteration of cellulose formation in tail regions of TALEN-electroporated specimens.

**Fig. 6:**
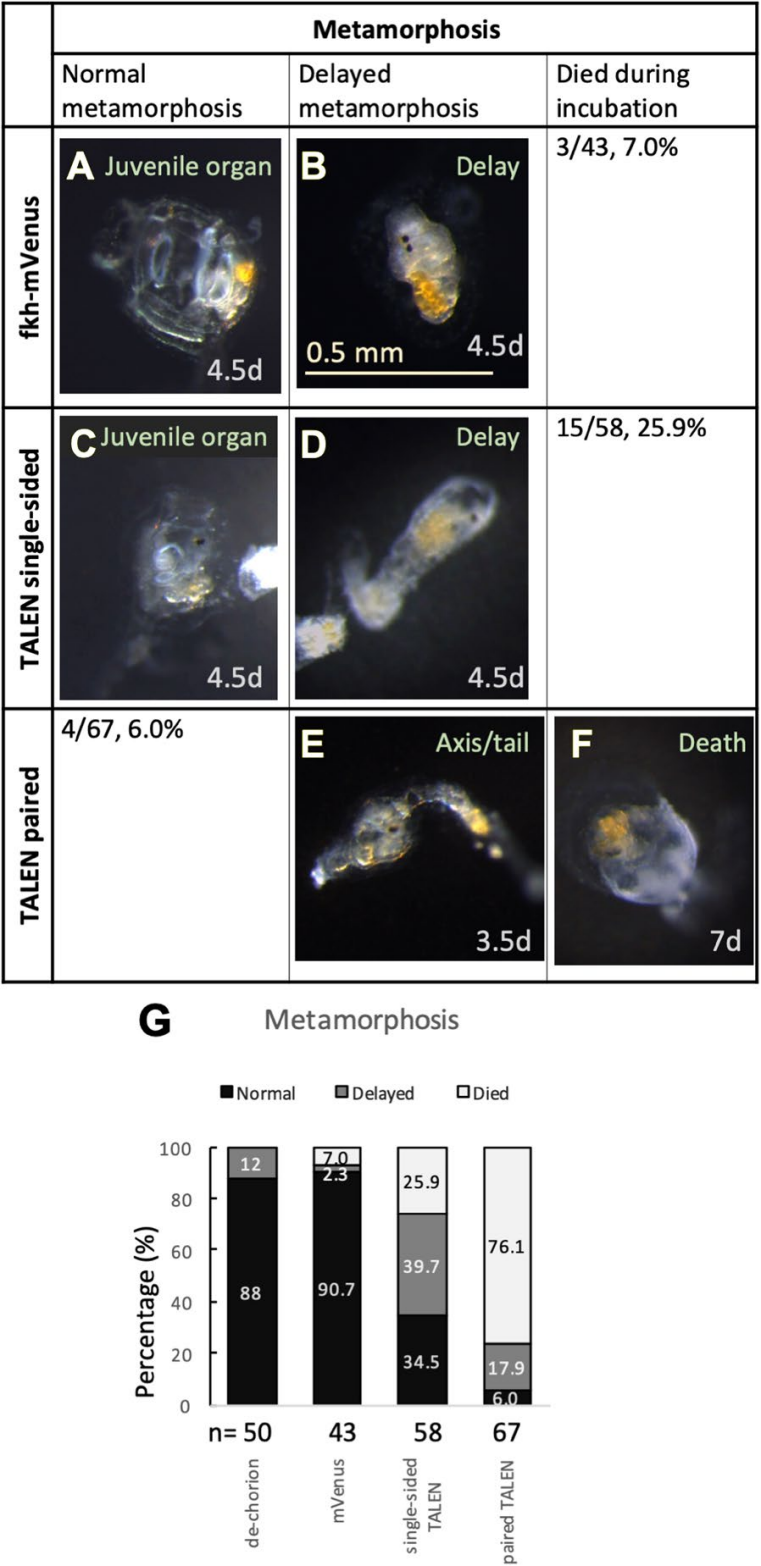
TALEN-mediated* GH6-1* knockout affected *Ciona* larval metamorphosis. **A**, **B** Control metamorphosing *Ciona* developed from eggs electroporated with mVenus plasmids. **A** A normally developing animal showing juvenile organs; **B** an individual showing successful resorption of the larval tail, but further metamorphosis steps were delayed. **C**, **D** Control metamorphosing *Ciona* developed from eggs electroporated with single-sided TALEN. **C** An individual that completed tail resorption, showing juvenile organs; **D** an animal showing incomplete tail resorption and delayed metamorphosis. **E**, **F** Experimental *Ciona* developed from eggs electroporated with paired TALEN plasmids. **E** An individual showing trunk axis rotation, but incomplete tail resorption; **F** an individual showing no progress of metamorphosis and dying cells. **G** Percentages of animals of each phenotype. Metamorphosis normal: on the sixth day post fertilization (6dpf), this juvenile had absorbed the tail, completed axis rotation, and showed at least one juvenile structure: mouth, gills, or endostyle. Metamorphosis delayed: at 6dpf, attached larvae either did not grow structures mentioned above or had not finished tail absorption. Three and half days to seven days post fertilization is represented as 3.5d, 4.5d, and 7d. The scale in **B** applies to all panels

TALEN-treated larvae showed a reduced metamorphosis rate (Fig. 6). Although many animals affected by both single-sided and paired TALEN showed abnormal tails (Additional file 1: Fig. S2) and were not able to swim normally, most larvae (> 90%) could still attach to the bottoms of petri dishes and started some degree of metamorphosis. Here, we define a successfully metamorphosed juvenile based on the following attributes: on the sixth day post fertilization, animals finished axis rotation, absorbed their larval tails, and formed at least one juvenile structure (mouth, gills, or the endostyle). Non-TALEN control specimens had a high metamorphosis rate (Fig. 6G, 88% for the dechorionated group and 90.7% for the mVenus group). TALEN-expressing animals showed a low rate of successful metamorphosis. The success rate of metamorphosis for single-sided TALEN-expressing specimens was reduced to 34.5% and that of paired TALEN-expressing specimens was only 6.0% (Fig. 6G). In addition, paired TALEN-electroporated larvae showed higher mortality (76.1%) than the single-sided TALEN group (25.9%). As previously stated, the cause of abnormal development of the single-sided TALEN group is not yet fully understood. However, specimens electroporated with the paired TALEN treatment showed a lower metamorphosis rate and higher death rate. Therefore, it is highly likely that expression of *GH6-1* is essential to regulate *Ciona* metamorphosis.

Other observed anomalies may result from overexpression of TALEN(s). Aside from the phenotypes mentioned above, many larvae with introduced TALEN, both in the single-sided group or in the group with full (paired) nucleases, showed abnormal epidermis (Additional file 1: Fig. S1, 52.4% for single-sided TALEN and 60.4% for paired TALEN). Epidermal cells were either rounder/thicker than normal cells (Fig. 5J) or showed irregular shapes (Additional file 1: Fig. S1B). Also, most TALEN-expressing larvae had curly tails (Additional file 1: Fig. S2, 76.2% for single-sided TALEN and 88.7% for paired TALEN). Swimming of these larvae was greatly impaired. As anomalies of epidermis and tail were found in both single-sided and paired TALEN groups with similar frequencies, it is likely that overexpression of exotic genes was responsible for these phenotypes.

## Discussion

Glycosyl hydrolases (also called glycoside hydrolases) are widely distributed enzymes that hydrolyze glycosidic bonds. Glycosyl hydrolases are essential to animal physiology. Examples include amylase [30, 31] for digestion, chitinase [32, 33] for immunity and digestion, lysozymes [34, 35] for anti-bacterial defense, and hyaluronidase [36, 37] for fertilization and bee venom. Based on amino acid sequence similarities, glycosyl hydrolases are grouped into families. As of February, 2023, there are 174 families of glycosyl hydrolases in the Carbohydrate Active Enzymes database [38–41]. Meanwhile, enzymes of a given family with similar sequences may acquire new catalytic specificities [38, 40]. The role of glycosyl hydrolases (cellulase) in cellulose production has not been explored yet, although one report showed that another cellulase, KORRIGAN, is a component of the cellulose synthesis complex of plants and can regulate growth of cell walls [42].

Our previous study showed that in addition to the GH6 domain of tunicate cellulose synthase (CesA), the *Ciona* genome contains a gene that encodes another GH6 domain-containing protein, named *GH6-1* [4, 16]. Molecular phylogeny demonstrated that as in the case of *CesA, GH6-1* was very likely acquired by the tunicate ancestor by horizontal gene transfer and has been maintained for more than 500 million years by extant tunicates [16]. Since *CesA* is expressed and functions in *Ciona* embryos, *GH6-1* is also expected to be expressed and to exhibit some function(s). As shown here, *GH6-1* is expressed in epidermal cells of tailbud embryos and early larvae, and is downregulated in late larvae. This expression pattern is quite similar to that of *CesA,* suggesting dynamic and coordinated expression regulation of the two genes. Expression control of *CesA* shows that one of the earliest zygotic transcription factors, Tfap2 (AP2), binds to the AP2 binding site between nucleotides -431 and -450 to activate *CesA* in epidermis of tailbud embryos [18, 19]. Whether *GH6-1* expression is controlled by Tfap2 (AP2) is an intriguing subject for future studies. On the other hand, scRNA analysis indicates that at the individual cell level, not all epidermal cells express the two genes coincidently. This suggests another regulator module(s) for *GH6-1*, which differs from the AP2 system of *CesA*. Different chromosomal locations of *CesA* (chromosome 7) and *GH6-1* (chromosome 3) suggest that it is unlikely that prokaryotic *CesA* became duplicated, with one duplicate retaining the original form of *CesA* and the other changing into *GH6-1.* In other words, horizontal transfers of prokaryotic *CesA* and *GH6-1* were likely independent and were recruited into different gene regulatory networks that forced expression in embryonic epidermis.

Epidermal expression at embryonic/larval stages of *Ciona GH6-1* implies a function different from digestion, as *Ciona* larvae do not have functional mouths and do not eat food particles [43, 44]. *GH6-1* expressing cells are enriched in the epidermis of tail tip, tail dorsal and ventral midlines, and papillae primordia. Coincidently, tissues expressing *GH6-1* correspond to structures that protrude outward: the larval tail elongates, the larval tail tunic expands outward in dorsal and ventral directions to form the larval fin [14], and adhesive papillae include protruding cells [45]. It is possible that GH6-1 hydrolase activity digests extracellular cellulose and softens the tunic; therefore, GH6-1 may permit extension/elongation of these structures. Beside common developmental biology methods, biochemical assays, for example, testing enzymatic activity of tunicate GH6-1 with purified recombinant proteins, will also be required to support this conjecture.

Cellulose metabolism is important to tunicate biology [14, 46]. *CesA* is essential in cellulose biosynthesis, but is also likely essential in development, especially in metamorphosis of swimming larvae to sessile juveniles [14], Nakashima et al., unpublished data). Disruption of *CesA* expression with an inserted transposon in the *swimming juvenile* mutant line resulted in lack of cellulose microfibrils and an abnormal tunic, but also in failure of normal metamorphosis [14]. In a larvacean tunicate, *Oikopleura dioica*, knockdown of *Od-CesA1* also resulted in anomalies in tail elongation, notochord cell alignment, and tailbud embryo hatching [13]. These observations demonstrate that cellulose metabolism contributes to processes other than tunic formation. Interestingly, we showed here that knockout of *Ciona GH6-1* resulted in a low rate of successful metamorphosis. Therefore, it is likely that both *CesA* and *GH6-1* participate in embryogenesis and metamorphosis of tunicates. While it is already known that *CesA* is required in normal *Ciona* embryonic and larval development, these developmental roles of *CesA* becomes feasible only with regulation or modification of *GH6-1*. Therefore, abnormalities of *GH6-1-*knockout larvae were likely caused by loss of the normal integrative function of CesA*,* which would occur when CesA interacts with GH6-1.

In this study, TALEN-mediated knockout experiments showed obvious morphological side effects in the single-sided TALEN-electroporation group. In hindsight, there are several ways to improve control experiments. First, to clarify whether phenotypes are due to dosage effect (too much TALEN nuclease product in the cells), a reduced amount of DNA used for electroporation will be considered. Alternatively, microinjection of in vitro*-*transcribed mRNA is another approach [47–49]. These methods could still knockout the target gene and produce fewer side effects. Second, instead of using a ubiquitously active promoter (EF1a in this study), using a tissue-specific promoter to limit TALEN expression in the tissue of interest should also reduce side effects [49]. To study epidermally expressed *GH6-1* and *CesA*, the promotor of *EpiI* would be a good candidate. Third, preparing other TALEN pairs in parallel could help to control potential off-target genomic cleavage. These TALENs would target either another gene that is not expressed in epidermis or a sequence that does not exist in the *Ciona* genome. In addition to effects of TALEN, it is also known that the popular dechorionation treatment in *Ciona* research affects left–right asymmetry [50] and the speed of tail resorption during metamorphosis [51]. Therefore, interpreting experimental phenotypes should be undertaken with care.

Although many questions remain to be answered, the present study unambiguously shows that another horizontally transferred gene, *GH6-1*, is expressed in embryonic epidermis and participates in metamorphosis of tunicate larvae. Lancelets (cephalochordates) and teleosts (vertebrates) exhibit relatively direct post-embryonic development. That is, overall outer morphology of larvae does not change drastically during metamorphosis. In contrast, metamorphosis of ascidian tunicates causes a dynamic change of swimming fish-like larvae into sessile juveniles with different morphology. Larvaceans, including *O. dioica*, do not show this kind of swimming-to-sessile transition, and GH6-1 in *O. dioica* shows a replacement of the conserved aspartic acid at the catalytic site [16]. If horizontally transferred genes are involved in tunicate metamorphosis, it seems highly likely, this is a unique evo-devo event that led to the tunicate lineage. In addition, since not only cellulose synthase, but also glycosyl hydrolases (cellulase) are involved in this process, tunicate *CesA* and *GH6-1* provide an experimental system to explore how horizontal gene transfer is involved in evolution of metazoan taxa.

## Conclusions

Tunicate *GH6-1*, a gene that originated by horizontal gene transfer of a prokaryote gene, was recruited into the ascidian genome, where it is expressed and functions in epidermal cells of embryos. Although further research is required, this observation demonstrates that both *CesA* and *GH6-1* are involved in evolution of unique tunicate cellulose metabolism, which shapes tunicate morphology and ecology.

## Methods

### Animal acquisition, fertilization, and culture of embryos

We obtained adult *Ciona intestinalis* type A from the National BioResource Project (NBRP) at Kyoto University with support of the Ministry of Education, Culture, Sports, Science and Technology, Japan. After arrival, animals were kept in the dark and fed with diatoms, *Chaetoceros calcitrans*, in small bucket of seawater overnight. The concentrated diatom product ‘sun culture’, purchased from Nisshin Marinetech (Yokohama, Kanagawa, Japan), was used. For each 20 animals, 30–100 mL of diatoms were given. Then animals were transferred to an aquarium, with seawater maintained at 18 °C. The aquarium was kept in continuous lighting to stimulate gamete production [52] and to prevent autonomous gamete spawning [53].

Seawater (FSW) used for culture of embryos was filtered with 0.22-µm polyethersulfone vacuum filters (Sartorius). Eggs and sperm were obtained surgically [54]. After insemination, eggs were dechorionated with FSW containing 0.1% w/v Actinase E (protease E) and 1% sodium thioglycolate, adjusted to pH 10. Dechorionated specimens were washed with FSW, and kept in FSW that contained 50 mg/L of streptomycin (streptomycin-seawater) in agarose-coated petri dishes and cultured at 18–20 °C.

### Reverse-transcription quantitative PCR

To determine *GH6-1* expression as well as *CesA* expression in *Ciona* embryos and larvae*,* total RNA was extracted from eggs, embryos, larvae, and young juveniles. Fifty to 100 individuals were collected in microcentrifuge tubes with minimal seawater (less than 100 µL). Then depending on the sample volume, samples were mixed with 600–1000 µL of TRIzol reagent (Invitrogen-Thermo Fisher Scientific). Eggs and embryos were homogenized by 15-s vortexing and hatched larvae and metamorphosing juveniles were manually ground with a BioMasher II homogenizer (Nippi Inc., Tokyo, Japan). Total RNA was extracted following manufacturer’s TRIzol protocol. Extracted total RNA was reverse transcribed to complementary DNA (cDNA) using a PrimeScript RT reagent Kit Perfect Real Time (Takara Bio, Shiga, Japan).

Dual-labeled fluorescein amidite–tetramethylrhodamine (FAM-TAMRA) probes and primer pairs for quantitative PCR (qPCR) were designed and synthesized by TaKaRa Bio, based on the following reference sequences: NCBI reference sequence XM_002131188 for *GAPDH* (glyceraldegyde-3-phosphate dehydrogenase), NM_001047983 for *CesA*, and XM_002119543 for *GH6-1,* respectively. For qPCR reactions, complementary DNA, probe-primer sets, and Premix Ex Taq polymerase mix (TaKaRa Bio) were prepared following the manufacturer’s protocol and processed on a StepOnePlus thermal cycler (Applied Biosystems, Thermo Fisher Scientific). The expression level of *GAPDH* was used for normalization.

### Whole-mount in situ hybridization

Fixation and whole-mount in situ hybridization of *Ciona* samples followed a protocol published by [55] with minor modifications, as follows. *Ciona* embryos, larvae, and young juveniles were fixed in 4% paraformaldehyde dissolved in MOPS buffer (0.1 M 3-Morpholinopropane-1-sulfonic acid, 0.5 M NaCl, pH 7.5) either overnight (12–16 h) at 4 °C or 1 h at room temperature. After fixation, samples were washed with phosphate-buffered saline and then with 75% ethanol. They were stored in 75% ethanol at -30 °C until use. Staging of embryos followed the tunicate anatomical and developmental ontology website TUNICANATO with related publications [44, 56, 57].

Antisense ribonucleic acid probes (riboprobes) were synthesized as follows. Total RNA of neurulae, tailbud, and larvae were extracted together with TRIzol (Invitrogen-Thermo Fisher Scientific) and reverse-transcribed to complementary DNA using SuperScript III First-Strand Synthesis SuperMix (Thermo Fisher Scientific). Then, cDNA mixtures were used to specifically amplify a few cDNA fragments (size 800–1100 bp) of the hybridization target genes via PCR. Amplicons were cloned into either pCR Blunt II TOPO vector (Invitrogen-Thermo Fisher Scientific) or pGEM T-Easy vector (Promega). Amplified cDNA was used as templates for digoxigenin-labeled riboprobe synthesis using DIG RNA labeling mix (Roche Diagnostics) and T7 or SP6 RNA polymerase (Roche Diagnostics). Sense strand riboprobes were also synthesized for control experiments.

For in situ hybridization, embryos were rehydrated with phosphate buffered saline (PBS), treated with proteinase K, post-fixed, treated with triethanolamine and acetic anhydride following the protocol [55] before 1 h of pre-hybridization. Hybridization of riboprobes at 42 °C was extended from 18 to 30 h. After anti-digoxigenin-antibody incubation, samples were washed 10 times in PBST (0.1% Tween-20 in phosphate-buffered saline). BM purple solution (Roche Diagnostics) was diluted [50% v/v, in alkaline-phosphatase buffer [55], pH 9.5] before being applied as a color development substrate. After color development, samples were washed in EDTA-PBS (10 mM EDTA in phosphate buffered saline to stop the enzymatic reaction. Then, they were washed in 30%, 50%, and 75% ethanol, rehydrated with PBS, and mounted in 70% Glycerol/30% PBS before imaging.

Sample images were recorded with an AxioCam HRc camera mounted on an Axio Imager Z1 microscope (Zeiss). To make signal identification easy, brightness of some images was adjusted, without creating image artifacts, using Fiji/ImageJ software [58]. For some embryos in which signals were distributed at different depths, the Extended Depth of Field plugin [59] of Fiji was applied to combine images of different depths.

### Single-cell RNA-seq data analysis

Cao et al. [26] reported and provided a publicly available single-cell transcriptome database of *Ciona* embryos and larvae (accession number GSE131155 on Gene Expression Omnibus). Using this database, we examined whether expression of *GH6-1* and *CesA* occurs in identical individual cells of developing *Ciona* embryos. Raw read FASTQ files were processed with CellRanger software (10X Genomics) to generate cell identifier barcodes and a gene read matrix based on a reference genome and KY gene models version 2019 [23].

Then, we used the Seurat toolkit version 3.2.1 [60] in RStudio version 1.2.5019 [61] to analyze gene expression of a late tailbud stage I sample (corresponding to Gene Expression Omnibus accession: GSM3764780). Low-quality droplets/cells were excluded based on a published method [26]: (1) cells with fewer than 1000 expressed genes were discarded, (2) only genes that were expressed in at least 3 cells were retained, (3) cells with unique molecular identifiers (UMIs) of five standard deviations above the mean were excluded. Cells that passed these filtering criteria (5034 cells) were kept. Expression of each cell was log-normalized. Genes with the 1000 highest standard deviations were designated as highly variable genes and used in principal component analysis. We selected dimensions 1 to 20 of the principal component analysis result for the graph-based clustering approach of Seurat and clustered partitioned cells into 30 clusters. Gene expression was examined by various plotting methods in the Seurat package: violin plot, FeatureScatter, and FeaturePlot.

### Gene knockout by TALEN-mediated genome editing

To examine possible physiological functions of *GH6-1* in *Ciona* embryos and larvae, we assembled two sets of transcription activator-like effector nuclease (TALEN) pairs following published methods [62, 63], and TALEN assembly protocols released on the NBRP-Ciona intestinalis Transgenic line RESources (NBRP-CITRES) website. A Platinum Gate TALEN Kit was acquired from the Addgene plasmid repository. To express TALEN in *Ciona*, an EF1a > TALEN-NG::2A::mCherry vector provided by Dr. Yasunori Sasakura (see also the NBRP-CITRES website) was used for the second-step assembly target. Optimal TALEN-binding targets in coding the sequence of *Ciona GH6-1* gene were selected with the assistance of TAL Effector Nucleotide Targeter 2.0 [64, 65]. Customized TALENs were designed to excise and disrupt the coding part of the *GH6-1* gene. One TALEN pair (TALEN no. 1) targeted CGGC-CTAC-TGAA-GGTC-T and ATTT-CGAA-CTGG-GATT (spanning the 394–442nd nucleotides of assumed coding sequence), and a second TALEN pair (TALEN no. 2) targeted TTCG-AACT-GGGA-TTAT and ATTT-CTAC-CTGG-ACAG (429–478th nucleotides). Since these targets cover upstream of the probable active site of *Ciona* GH6-1 protein [16], these TALEN pairs were expected to disrupt the function of *Ciona* GH6-1 protein.

TALEN-encoding plasmids were introduced to dechorionated-fertilized eggs by electroporation following a protocol [63]. Dechorionation steps were applied to unfertilized eggs before the insemination step. After seawater washing, eggs were fertilized, and then dechorionated-fertilized *Ciona* eggs with minimal filtered seawater were added to mannitol solution (0.77 M mannitol in 10% v/v filtered seawater). Then, for each electroporation group, plasmid DNA (dissolved in 80 µL of TE buffer) was combined with 720 µL of the aforementioned egg-mannitol mix, making a final 800-µL volume for each electroporation group. Electroporation (50 V, 20 ms, in 4-mm cuvettes) was performed with a GenePulser Xcell pulser (Bio-Rad). After pulsing, eggs were carefully transferred into streptomycin-seawater in agarose-coated or gelatin-coated plastic petri dishes and incubated at 18 °C. In the control group, a plasmid containing a promotor of the *Ciona* forkhead gene (*Ci-fkh*, also known as *Ci-FoxA-a*) and monomer Venus fluorescent protein gene (a generous gift from Dr. Koki Nishitsuji of our research unit) was used. In TALEN groups, 20 to 60 µg of DNA (10 to 30 µg for each plasmid of two units of a TALEN pair) were used in one experiment. Only one TALEN plasmid was used in the single-sided TALEN control group. After electroporation, eggs were transferred to streptomycin-seawater and incubated at 18 °C.

A few embryos were used for checking the specific editing efficiency. Genomic DNA of 15–30 embryos (12–16 hpf) was extracted with a Maxwell RSC Blood DNA Kit on a Maxwell RSC Instrument (Promega). Extracted genomic DNA was amplified with primers targeting a part of the *GH6-1* gene; Forward: 5′-GCC TCG CTA CAA GAA CCA CC and Reverse: 5′-ACA CAA TGA CTT TTC GAG CGC. Amplicons were purified, cloned into pGEM-T-Easy vector (Promega), and sequenced with BigDye Terminator v3.1 kit on SeqStudio Genetic Analyzers (Thermo Fisher Scientific). Sequences of paired TALEN-electroporated genomic DNA amplicons were compared with the corresponding part of untreated GH6-1 gene sequence. Amplicons edited by TALEN showed insertion or deletion of 1 to 23 nucleotides.

Electroporated embryos were examined after reaching the neurula stage (about 10 hpf) under a Leica M205-FA microscope. Embryos that actually received and expressed the introduced plasmid showed the red fluorescence of mCherry protein, and only these embryos were used subsequently.

Crystallized cellulose of manipulated *Ciona* larvae was stained for microscopic examination following the method of [11] with a few modifications. Commercial green fluorescent protein-tagged carbohydrate-binding module (Carbohydrate Binding Module 3A, GFP-CBM3, origin: *Bacteroides cellulosolvens*) was purchased from NZYtech. The protein was centrifuged and re-dissolved in an assay buffer (20 mM Tris–HCl, pH 7.5, 20 mM NaCl, 5 mM CaCl_2_) according to the manufacturer’s protocol. It was diluted to 1/6 concentration in assay buffer before application. *Ciona* embryos or larvae were fixed and preserved as described above. These samples were rehydrated in three washes (10 min each) of phosphate buffered saline containing 0.1% Tween-20 (PBSTw), incubated in blocking solution [PBS with 1% Blocking Reagent (Roche)] for 1 h at room temperature, washed two times in Tris-buffered saline containing 0.1% Tween-20, rinsed twice in assay buffer, stained with diluted GFP-CBM3 assay solution for 12–20 h at 4 °C, washed 8 times in Tris-buffered saline + 0.1% Tween-20 buffer, and transferred to VECTASHIELD Antifade Mounting Medium (Vector Laboratories) before microscopic imaging.

## Supplementary Information

**Additional file 1: Figure S1.** Abnormal epidermis in TALEN-impacted larvae. Among larvae impacted by TALEN plasmids, either single-sided or a full pair. Some show normal epidermis and elongated tails (A). Many larvae show abnormal epidermis (B). Refer to Figure S2 for tail anomalies. (C) Abnormal epidermis: rounder/thicker/irregular epidermal cells. **Figure S2**. Many TALEN-affected *Ciona* larvae showed abnormal tails. (A, B) Control larvae developed from eggs electroporated with mVenus plasmids. A, normally hatched larva with a well elongated tail. B. Some larvae had curly tails or tails that failed to elongate. (C, D) Control larvae developed from eggs electroporated with single-sided TALEN. C. A newly hatched larva with an elongated tail. D. Larvae with curly tails. (E, F) Experimental larvae developed from eggs electroporated with paired TALEN plasmids. E. A newly hatched larva with an elongated tail. F. Some larvae showed curly tails. (G) Percentage of tail phenotypes. 18h: 18 h post fertilization. 1d: one day post fertilization. The scale in B applies to all panels.

## Data Availability

Not applicable.
