# Supplementary material for: Expression and possible functions of a horizontally transferred glycosyl hydrolase gene, GH6-1, in Ciona embryogenesis
Source: EvoDevo. 2023 Jul 11;14:11. doi: 10.1186/s13227-023-00215-x (PMC10334666; doi:10.1186/s13227-023-00215-x)
Supplement: Supplementary file 1 — Additional file 1: Figure S1. Abnormal epidermis in TALEN-impacted larvae. Among larvae impacted by TALEN plasmids, either single-sided or a full pair. Some show normal epidermis and elongated tails (A). Many larvae show abnormal epidermis (B). Refer to Figure S2 for tail anomalies. (C) Abnormal epidermis: rounder/thicker/irregular epidermal cells. Figure S2. Many TALEN-affected Ciona larvae showed abnormal tails. (A, B) Control larvae developed from eggs electroporated with mVenus plasmids. A, normally hatched larva with a well elongated tail. B. Some larvae had curly tails or tails that failed to elongate. (C, D) Control larvae developed from eggs electroporated with single-sided TALEN. C. A newly hatched larva with an elongated tail. D. Larvae with curly tails. (E, F) Experimental larvae developed from eggs electroporated with paired TALEN plasmids. E. A newly hatched larva with an elongated tail. F. Some larvae showed curly tails. (G) Percentage of tail phenotypes. 18h: 18 h post fertilization. 1d: one day post fertilization. The scale in B applies to all panels. [file 13227_2023_215_MOESM1_ESM.docx]

**Expression and possible functions of a horizontally transferred glycosyl hydrolase gene, *GH6-1*, in *Ciona* embryogenesis**

**Additional file**


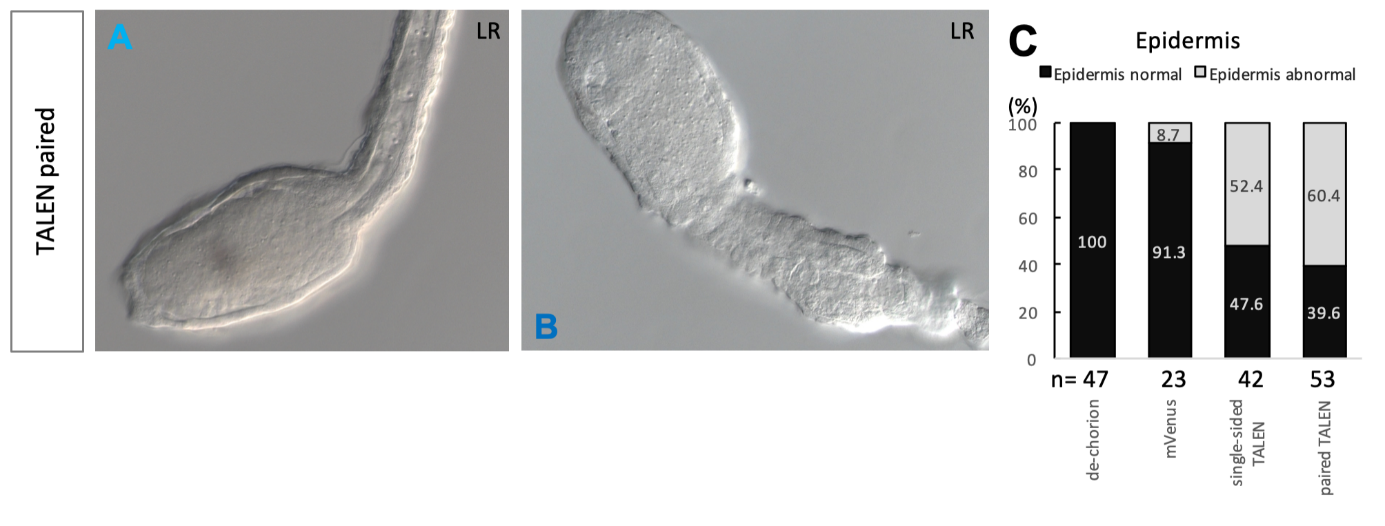


Figure S1. Abnormal epidermis in TALEN-impacted larvae.

Among larvae impacted by TALEN plasmids, either single-sided or a full pair. Some show normal epidermis and elongated tails (A). Many larvae show abnormal epidermis (B). Refer to Figure S2 for tail anomalies. (C) Abnormal epidermis: rounder/thicker/irregular epidermal cells.


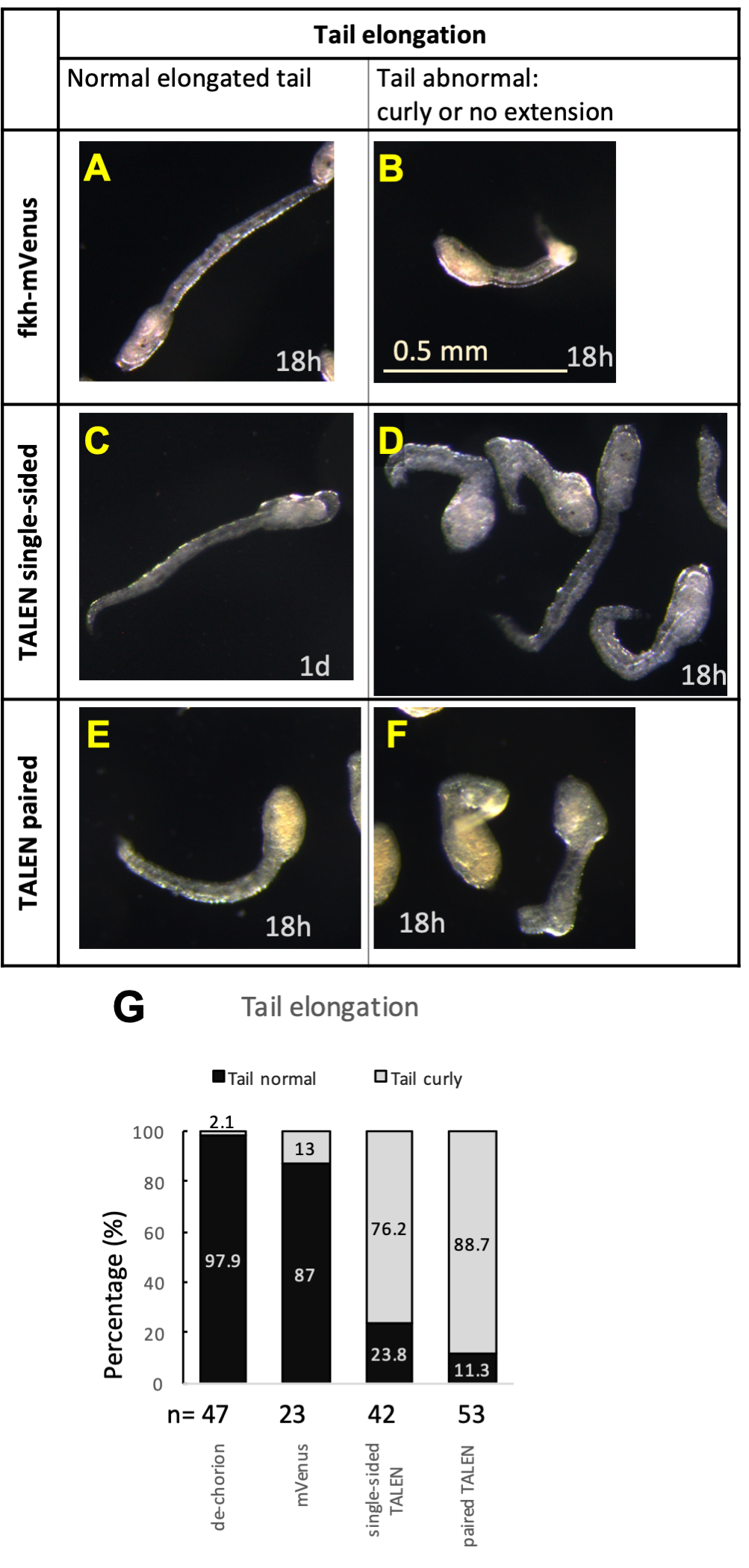


Figure S2. Many TALEN-affected *Ciona* larvae showed abnormal tails.

(A, B) Control larvae developed from eggs electroporated with mVenus plasmids. A, normally hatched larva with a well elongated tail. B. Some larvae had curly tails or tails that failed to elongate. (C, D) Control larvae developed from eggs electroporated with single-sided TALEN. C. A newly hatched larva with an elongated tail. D. Larvae with curly tails. (E, F) Experimental larvae developed from eggs electroporated with paired TALEN plasmids. E. A newly hatched larva with an elongated tail. F. Some larvae showed curly tails. (G) Percentage of tail phenotypes. 18h: 18 h post fertilization. 1d: one day post fertilization. The scale in B applies to all panels.
